# Homocysteine level is positively and independently associated with serum creatinine and urea nitrogen levels in old male patients with hypertension

**DOI:** 10.1038/s41598-020-75073-x

**Published:** 2020-10-22

**Authors:** Qianhong Yang, Youwei Lu, Yanhua Deng, Jiayi Xu, Xi Zhang

**Affiliations:** Department of Geriatrics, Minhang Hospital, Fudan University, 170 Xinsong Road, Minhang District, Shanghai, 201199 People’s Republic of China

**Keywords:** Nephrons, Predictive markers

## Abstract

A cross-sectional study to show whether and how serum fasting homocysteine levels are associated with renal function changes in patients with hypertension. Homocysteine levels were associated with serum creatinine and blood urea nitrogen (BUN) levels with coefficients of 2.04 and 0.07, respectively, only in males and independent of confounders. In addition, low density lipoprotein cholesterol (LDL-C) levels were positively and left ventricular ejection fraction (LVEF) was negatively associated with serum creatinine level in males; age was positively associated with serum creatinine levels in females. Age was a common risk factor positively associated with BUN levels in both sexes, while total cholesterol (TC) levels and glycemic control were independent risk factors that were positively associated with BUN levels only in males. LDL-C levels and LVEF were negatively associated with BUN levels in females. Body mass index (BMI) was positively associated and hemoglobin A1c (HbA1c) levels, high density lipoprotein cholesterol (HDL-C) levels and the presence of stroke were negatively associated with serum uric acid levels in male patients. In contrast, only LVEF was positively associated with uric acid levels in females. In conclusion, homocysteine level is an independent risk factor associated with serum creatinine and BUN levels in male patients with hypertension.

## Introduction

Homocysteine is a sulfur-containing, nonproteinogenic amino acid that is derived from transmethylation of the essential sulfur-containing amino acid methionine^1^. Homocysteine can be either degraded through the transsulfuration pathway or remethylated to methionine^2,3^. In the transsulfuration pathway, homocysteine combines with serine to form cystathionine; then, cystathionine is hydrolyzed into cysteine and alpha-ketobutyrate^2^. There are two different remethylation pathways: the first remethylation pathway requires 5-methyltetrahydrofolate as a methyl donor, which is generated by a reaction catalyzed by 5,10-methylenetetrahydrofolate reductase (MTHFR); the second remethylation reaction uses betaine as a methyl donor in the transsulfuration pathway^2,3^. Both remethylation reactions are irreversible and require the active form of B vitamins as a cofactor^4,5^. Homocysteine is filtered by the glomerulus; however, it is almost completely (over 98%) reabsorbed by tubular cells in the kidneys^3^.

The normal total fasting plasma homocysteine levels range from 4 to 12.3 μmol/l^6^. Hyperhomocysteinemia is currently defined as a homocysteine level above 10 μmol/l in the blood and is demonstrated as a pathological condition^6,7^. Currently, the hypothesis focused on homocysteine toxicity can be summarized as homocysteinylation (protein structure modifications); oxidative stress induction; and excitotoxicity^1^. These biotoxicities are believed to act in numerous diseases^1^. Hyperhomocysteinemia has been recognized to be associated with cardiovascular diseases (CVDs)^8^, neurological and psychiatric disorders^9^, bone tissue damage^10^, gastrointestinal disorders^11^, cancer^12^, and congenital disorders as well as chronic kidney disease (CKD)^1,13^. In addition, the prevalence of hyperhomocysteinemia is high in patients undergoing peritoneal dialysis and in renal transplantation recipients^4^.

The existence of a narrow crosstalk between homocysteine, hypertension and renal disease in pathophysiological processes has increased the difficulty of research attempting to identify the pathogenesis of hyperhomocysteinemia. Homocysteine is an independent risk factor for both hypertension and CKD; at the same time, hypertension is also a well-documented cause of CKD^2^. However, studies focused on the correlations between homocysteine levels and renal disorders remain scarce^14^, and the existing research differs in the study design, objective, setting and participants. Although observational studies have shown that hyperhomocysteinemia is associated with the risk of developing CKD and albuminuria in the general population^5,14–17^, reports showed that elevated serum homocysteine levels are not correlated with serum uric acid levels in patients with gout^18^.

China is a country where no folic acid fortification is implemented. Hypertension and hyperhomocysteinemia often presented together (termed H-type hypertension), which accounts for approximately 75% of patients with hypertension^19–21^. Chinese clinicians believe that H-type hypertension is correlated with a higher occurrence of stroke in Chinese adults with hypertension^22^. However, how serum homocysteine levels are associated with renal function in Chinese patients with hypertension remains unclear. In this report, this issue was evaluated in 323 male and 177 female hypertension patients.

## Methods

### Study design and participants

**Figure 1:**
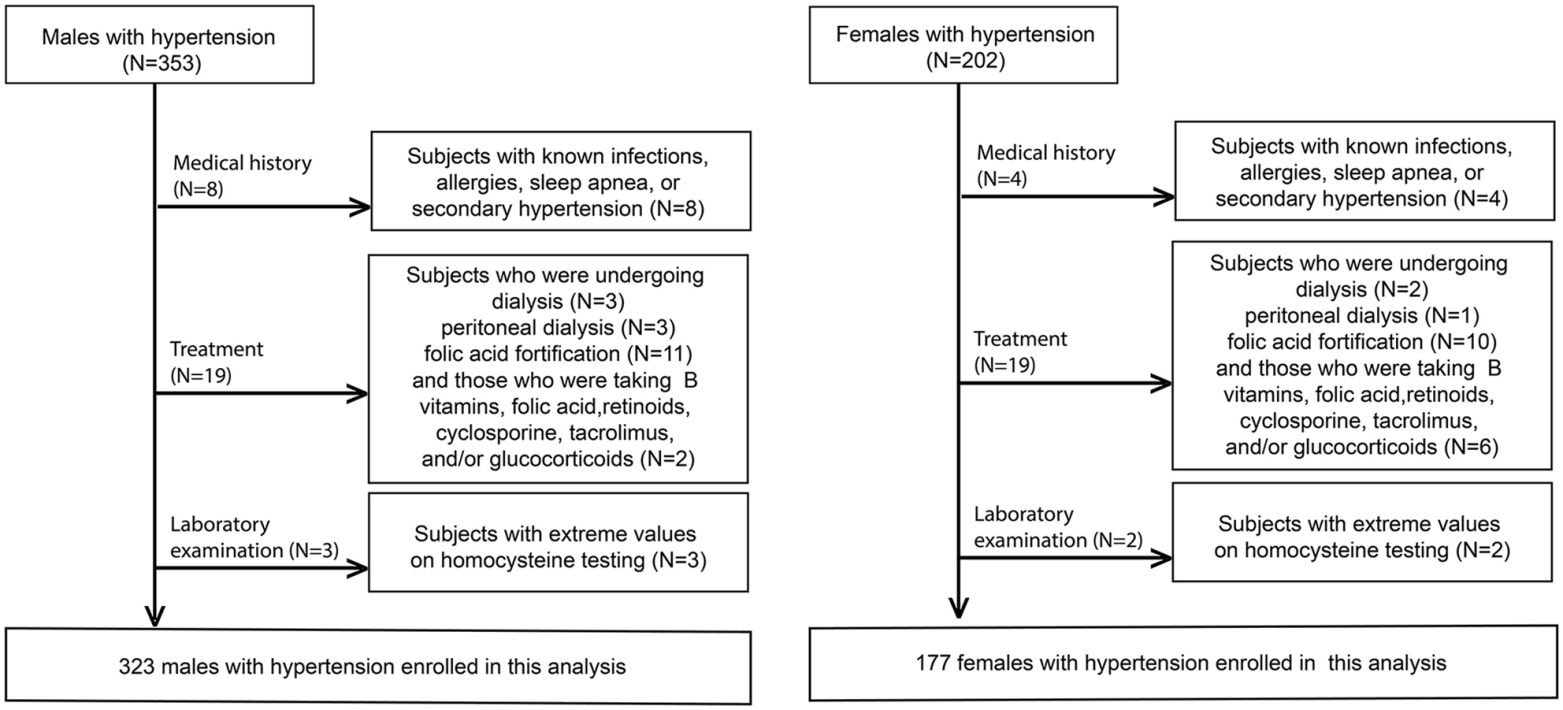
Flow chart of participants. In total, 353 males and 202 females were involved initially. 30 males and 25 females were excluded based on exclusion criteria. Finally, 323 males and 177 females were involved in this study.

This is a cross-sectional study to show whether and how serum fasting homocysteine levels are associated with renal function indicators in old Chinese patients with hypertension. To derive more information from the relationship between homocysteine level and renal function, we have not used a surrogate marker eGFR and instead used serum creatinine, BUN and uric acid levels as renal function indicators directly. Initially, 353 male and 202 female registered hypertensive inpatients in the Departments of Geriatrics in our hospital in 2019 were continuously assembled. Subjects who were undergoing dialysis (N = 5) or peritoneal dialysis (N = 4) or folic acid fortification (N = 21) and those who were taking B vitamins, folic acid, retinoids, cyclosporine, tacrolimus, and/or glucocorticoids (N = 8) were excluded, as were those with known infections, allergies, sleep apnea, or secondary hypertension (N = 12) and those with extreme values on homocysteine testing (> 40.0 μmol/l, N = 5) (Fig. 1). Finally, 323 males and 177 females were involved in this study.

The study was approved by the institutional review board at Minhang Hospital, Fudan University and was conducted in full accordance with the principles of the Declaration of Helsinki. All patients provided written informed consent.

### Data collection

Age, weight, height, smoking, alcohol consumption, history of hypertension, stroke, diabetes, CHD, CKD and medications were recorded using an admission registration questionnaire. Smoking was defined as smoking five or more cigarettes daily for at least 3 months. Alcohol consumption was defined as drinking up to one alcoholic drink per day for women and up to two per day for men irrespective of liquor types. Body mass index (BMI) was calculated as weight divided by height squared (kg/m^2^). Waking blood pressure was measured between the hours of 8 and 11 a.m. in the seated position after a 5-min rest. It was recorded as the mean of three measurements taken at 1-min intervals according to American Society of Hypertension guidelines and using a mercury sphygmomanometer. Hypertension was defined as a systolic pressure greater than 140 mm Hg or a diastolic pressure greater than 90 mm Hg or according to medical history.

A fasting blood sample of each participant was sampled from the antecubital vein the next morning after admission. Serum lipid profiles, HCY, folic acid, HbA1c creatinine, BUN, uric acid, and serum glucose concentrations were measured by the hospital laboratory according to routine procedures. Diabetes was defined as a fasting plasma glucose of at least 7.0 mmol/L or treatment with antidiabetic medication before the measurement.

A transthoracic echocardiographic examination was performed using a Sonos 5500 type Ultrasound machine (Philips, Best, Netherlands) with a 2.5-Hz transducer. The measurement of the left ventricular ejection fraction was performed using Simpson’s biplane method.

The diagnosis of CHD and CKD followed the 2019 ESC Guidelines for the Diagnosis and Management of Chronic Coronary Syndromes^23^. The Task Force for the Diagnosis and Management of Chronic Coronary Syndromes of the European Society of Cardiology (ESC) and Classification and Diagnosis of Diabetes: Standards of Medical Care in Diabetes-2020^24^.

### Statistical analysis

All variables generally fit a normal distribution as checked by histogram analysis after eliminating a small number of extremes (< 1% for each variate). Shapiro–Wilk test for three dependent variables, serum creatinine, BUN and serum uric acid, also confirmed their normality (p > 0.05). Thus, they are presented as the means ± standard deviations.

To learn whether and how serum creatinine, BUN and uric acid levels were associated with fasting serum homocysteine level, regression modeling was adopted. To eliminate the effect of multicollinearity on the results, internal correlations within parameters were quantified by calculating the Pearson correlation coefficient. The levels of serum creatinine, BUN and serum uric acid displayed high and significant correlations between each other; therefore, these variables were designated as dependent variate solely in each regression analysis. Age, BMI, levels of homocysteine, folic acid, creatinine, BUN, uric acid, TC, TG, LDL-C, and HDL-C, SBP, DBP, percentages of HbA1c and LVEF, presence of diabetes, CHD, CKD and stroke, and history of medication, smoking and drinking were treated as independent variates.

The associations between independent and dependent variates were evaluated by two steps, univariate and multivariate linear regressions. In univariate regression analysis, the associations between each independent variate and each dependent variate were evaluated, any independent variate that displayed p < 0.10 in a given univariate analysis model was considered as a potential independent variable.

In multivariate regression analysis, the independent variable selection was based on the preliminary univariate analysis, Pearson correlation coefficient and principles of biochemistry and physiology (detailed in the results); independent factors associated with renal function indicators were evaluated under adjustment of these confounding factors with backward selection. We considered *P* < 0.05 by a two-sided test to be indicative of statistical significance. Statistical analyses were performed using SPSS ver. 17.0.0 (SPSS, Chicago, IL, USA).

## Results

### Participants

As summarized in Table 1, the average ages of males and females were 75.6 ± 13.7 and 75.9 ± 11.3 years, respectively. The average BMI of males and females were 24.5 ± 3.0 and 24.3 ± 3.8 kg/m^2^, respectively, suggesting that most of our subjects had a normal to overweight BMI. The average HCY levels of males and females were 16.5 ± 7.1 and 13.6 ± 5.9 μmol/L, respectively, which indicates that the HCY levels of most subjects exceeded the upper bound. The average serum folic acid levels of males and females were 7.6 ± 4.6 and 9.2 ± 4.7 ng/mL, which shows that most of our subjects possessed a normal range of serum folic acid levels (2.7 to 17.0 ng/mL). Approximately 42.9% males and females had diabetes, and the average HbA1c levels of males and females were 6.8 ± 1.7 and 6.6 ± 1.6%, respectively, which is consistent with a high prevalence of diabetes among our subjects. Although only 13.4% of males and 7.3% of females were diagnosed with CKD and the average serum creatinine, BUN and uric acid levels were in the normal ranges, their renal function adjoined to the upper limits. Although all participants had hypertension, the average SBP and DBP for males and females were 138.9 ± 23.4 and 141.2 ± 19.4, respectively, and 78.2 ± 12.0 and 78.6 ± 10.7 mmHg, respectively. These measures were relatively low and might be due to medication since 75.8% of males and 78.5% of females were taking beta-blockers, angiotensin-converting enzyme inhibitors, angiotensin receptor blockers, statins and/or antidiabetic agents.

**Table 1:** Clinical characteristics of subjects.

Variable	Male	Female
	(N = 323)	(N = 177)
Average age, years	75.6 ± 13.7	75.9 ± 11.3
Age range, years	40.0–99.0	41.0–98.0
Lower quartile, median and upper quartile of age, years	66.0, 75.0, 87.0	66.0, 73.0, 84.0
BMI, kg/m^2^	24.5 ± 3.0	24.3 ± 3.8
HCY, µmmol/L	16.5 ± 7.1	13.6 ± 5.9
Folic acid, ng/mL	7.6 ± 4.6	9.2 ± 4.7
HbA1c, %	6.8 ± 1.7	6.6 ± 1.6
Creatinine, µmol/L	99.2 ± 72.1	76.8 ± 49.0
BUN, mmol/L	6.6 ± 3.4	6.0 ± 2.9
Uric acid, µmol/L	353.6 ± 103.8	312.1 ± 104.6
TC, mmol/L	3.9 ± 1.0	4.3 ± 1.1
TG, mmol/L	1.5 ± 1.0	1.6 ± 1.0
LDL-C, mmol/L	2.6 ± 0.9	2.8 ± 1.0
HDL-C, mmol/L	1.0 ± 0.3	1.2 ± 0.3
SBP, mmHg	138.9 ± 23.4	141.2 ± 19.4
DBP, mmHg	78.2 ± 12.0	78.6 ± 10.7
LVEF, %	62.9 ± 5.1	62.6 ± 5.0
Presence of diabetes, cases (%)	138 (42.9)	76 (42.9)
Presence of CHD, cases (%)	58 (18.0)	37 (20.9)
Presence of CKD, cases (%)	43 (13.4)	13 (7.3)
Presence of stroke, case (%)	184 (57.1)	85 (48.0)
Undergoing medication, cases (%)	244 (75.8)	139 (78.5)
Smoker (%)	111 (34.5)	3 (1.7)
Drinker (%)	53 (16.5)	2 (1.1)

Continuous variables were presented as means ± standard deviation (SD) and categorical data were presented as the number (percentage). Differences between groups were examined by using T test or χ2 tests according to the characteristics of data distribution.

*BMI* body mass index, *HCY* homocysteine, *HbA1c* hemoglobin A1c, *BUN* blood urea nitrogen, *TC* total cholesterol, *TG* triglyceride, *HDL-C* high density lipoprotein cholesterol, *LDL-C* low density lipoprotein cholesterol, *SBP* systolic blood pressure, *DBP* diastolic blood pressure, *LVEF* left ventricular ejection fraction, *HTN* hypertension, *CHD* coronary heart disease, *CKD* chronic kidney disease.

### Factors associated with serum creatinine level in the univariate regression analysis

To identify which clinical parameters are associated with renal function change, in the following analyses, serum creatinine, BUN and uric acid were treated as dependent variables, and the other parameters listed in the Table 1 treated as independent variables to perform the univariate regression analysis.

As shown in Table 2 (upper panel), serum HCY, BUN, uric acid, TC and LDL-C levels, SBP, and DBP (*P* = 0.074) were positively associated and LVEF was negatively associated with serum creatinine levels in male patients with hypertension. Age and serum HCY, BUN and uric acid levels were positively associated with serum creatinine level in female patients with hypertension; serum creatinine levels were higher in females with CHD and CKD and lower in those with stroke.

**Table 2:** Factors associated with renal function in the univariate regression analysis.

Variable	Male		Female	
	(N = 323)	*P*	(N = 177)	*P*
**Factors associated with serum creatinine level**				
Age, years	–	–	1.29 (0.67 to 1.90)	< 0.001
HCY, μmol/L	2.59 (1.51 to 3.67)	< 0.001	2.74 (1.57 to 3.92)	< 0.001
BUN, mmol/L	15.33 (13.74 to 16.92)	< 0.001	13.48 (12.02 to 14.95)	< 0.001
Uric acid, μmol/L	0.10 (0.03 to 0.18)	0.007	0.21 (0.14 to 0.27)	< 0.001
TC, mmol/L	10.21 (2.72 to 17.70)	0.008	–	–
LDL-C, mmol/L	10.58 (2.01 to 19.15)	0.016	–	–
SBP, mmHg	0.43 (0.09 to 0.77)	0.013	–	–
DBP, mmHg	0.60 (− 0.60 to 1.26)	0.074	–	–
LVEF, %	− 0.33 (− 0.46 to − 0.19)		–	–
Presence of CHD, cases (%)	–	–	1.01 (1.00 to 1.01)	0.099
Presence of CKD, cases (%)	–	–	1.10 (1.04 to 1.16)	< 0.001
Presence of stroke, case (%)	–	–	0.99 (0.98 to 1.00)	0.046
**Factors associated with serum BUN**				
Age, years	0.05 (0.02 to 0.07)	0.001	0.09 (0.05 to 0.12)	< 0.001
HCY, μmol/L	0.12 (0.07 to 0.17)	< 0.001	0.19 (0.13 to 0.27)	< 0.001
TC, mmol/L	0.31 (− 0.05 to 0.67)	0.091	–	–
LDL-C, mmol/L	–	–	− 0.37 (− 0.79 to 0.06)	0.093
DBP, mmHg	–	–	− 0.04 (− 0.08 to 0.00)	0.076
LVEF, %	− 0.09 (− 0.16 to − 0.01)	0.018	− 0.12 (− 0.21 to − 0.36)	0.006
Diabetes, cases (%)	1.17 (1.08 to 1.27)	< 0.001	–	–
Diabetes treatment, cases (%)	1.16 (1.07 to 1.25)	< 0.001	1.12 (1.00 to 1.24)	0.043
Presence of CHD, cases (%)	–	–	1.11 (1.00 to 1.24)	0.056
Presence of CKD, cases (%)	1.55 (1.36 to 1.77)	< 0.001	1.75 (1.38 to 2.21)	< 0.001
Presence of stroke, case (%)	0.94 (0.88 to 1.01)	0.076	0.86 (0.75 to 0.97)	0.016
Drinker (%)	0.87 (0.76 to 0.99)	0.032	–	–
**Factors associated with serum uric acid**				
Age, years	− 0.71 (− 1.54 to 0.12)	0.093	1.47 (0.11 to 2.83)	0.035
BMI, kg/m^2^	6.63 (92.81 to 10.45)	0.001		
HCY, μmol/L	2.28 (0.69 to 3.87)	0.005	3.42 (0.81 to 6.02)	0.011
HbA1c, %	− 0.10 (− 0.17 to − 0.04)	0.002	–	–
TC, mmol/L	11.31 (0.48 to 22.14)	0.041	–	–
TG, mmol/L	13.59 (2.43 to 24.75)	0.017	–	–
LDL-C, mmol/L	10.81 (− 1.59 to 23.21)	0.087	–	–
HDL-C, mmol/L	− 51.96 (− 91.49 to − 12.43)	0.011	− 39.14 (− 83.88 to 5.60)	0.086
LVEF, %	–	–	− 3.98 (− 7.11 to − 0.86)	0.013
Presence of diabetes, cases (%)	0.99 (0.99 to 1.00)	0.069	–	–
Presence of CKD, cases (%)	1.01 (1.01 to 1.01)	< 0.001	1.01 (1.01 to 1.02)	< 0.001
Presence of stroke, case (%)	1.00 (1.00 to 1.00)	0.012	1.00 (0.99 to 1.00)	0.019

For the abbreviations, please see Table 1. "–" indicates that a variable is not a potential independent one in a given univariate analysis model (p > 0.1). HCY was input into each model as continuous variable. When both dependent and independent variates were continuous variables, the associations were quantified by coefficients and 95% confidential intervals (95% CIs); when dependent variables were categorical variable, the associations were quantified by odds ratios (ORs) and 95% CIs.

### Factors associated with serum BUN level in the univariate regression analysis

As shown in Table 2 (middle panel), age and serum HCY and TC (*P* = 0.091) levels were positively associated and LVEF was negatively associated with serum BUN levels in male patients; serum BUN levels were higher in male patients with diabetes and CKD who had glycemic control; conversely, the BUN levels were lower in males with stroke (*P* = 0.076) and in males who consumed alcohol. Age and HCY levels were positively associated and LDL-C levels (*P* = 0.093), DBP (*P* = 0.076) and LVEF were negatively associated with serum BUN levels in female patients; serum BUN levels were higher in females who had glycemic control, CHD (*P* = 0.056) and CKD and were lower in those with stroke.

### Factors associated with serum uric acid level in the univariate regression analysis

As shown in Table 2 (lower panel), age (*P* = 0.093) and HbA1c and HDL-C levels were factors negatively associated and BMI and HCY, TC, TG and LDL-C levels (*P* = 0.087) were factors positively associated with serum uric acid levels in male patient with hypertension; serum uric acid levels were higher in those with CKD and stroke and lower in those complicated with diabetes (*P* = 0.069). Age and HCY levels were positively associated and HDL-C levels (*P* = 0.086) and LVEF were negatively associated with serum uric acid levels in female patients; serum uric acid levels were higher in females complicated with CKD and/or stroke.

### Selection of variables for the multivariate regression analysis

Strict quality control is one of the preconditions to ensure the reliability of multivariate analysis. In this report, the principles of biochemistry, clinical practice and Pearson correlation analyses were adopted comprehensively to be the basis for variable selection.

In general, serum creatinine, BUN and uric acid levels solely acted as dependent variables; HDL-C and LDL-C were selected to represent lipid profile; “SBP and DBP” and “the presence of diabetes and undergoing glycemic control” were input into the multivariate regression model individually to avoid multicollinearity.

Thus, “HCY, LDL-C, SBP/DBP and LVEF” and “age, HCY, presence of CHD, CKD and stroke” were variable sets for males and females in the multivariate regression analysis to identify the independent associations with serum creatinine levels. Age, HCY and TC levels, LVEF, undergoing glycemic control, the presence of CKD and stroke, and alcohol consumption were variable sets for males, and age, HCY and LDL-C levels, DBP, LVEF, undergoing glycemic control, the presence of CHD, CKD and stroke were variable sets for females to identify the independent associations with serum BUN levels. Age, BMI, HCY, HbA1c, LDL-C, and HDL-C levels and the presence of diabetes, CKD and stroke were variable sets for males, and age, HCY and HDL-C levels, LVEF, and the presence of CKD and stroke were variable sets for females to identify independent associations with serum uric acid levels.

### Factors independently associated with serum creatinine levels

As shown in Table 3, multivariate regression analyses showed that HCY and LDL-C levels were factors positively associated and LVEF was a factor negatively associated with serum creatinine levels independently in males with hypertension. Neither SBP nor DBP were independent factors associated with serum creatinine levels in males. In females with hypertension, only age and the presence of CKD remained as independent factors to predict serum creatinine levels; HCY levels, the presence of CHD and stroke lost independence in association with serum creatinine levels in females.

**Table 3:** Factors associated with serum creatinine levels in the multivariate regression analysis.

Variable	Male		Female	
	(N = 323)	*P*	(N = 177)	*P*
Age, years	–		0.48 (0.01 to 0.95)	0.043
HCY, μmol/L	2.04 (1.08 to 3.00)	< 0.001	–	–
LDL-C, mmol/L	10.26 (3.05 to 17.48)	0.005	–	–
LVEF, %	− 3.00 (− 4.32 to − 1.70)	< 0.001	–	–
Presence of CKD, cases (%)	–	–	127.39 (107.20 to 147.57)	< 0.001

For the abbreviations, please see Table 1. Associations are expressed as coefficients and 95% CIs. "–" indicates that a variable is not an independent one in a given multivariate analysis model (p > 0.05).

### Factors independently associated with serum BUN levels

As shown in Table 4, multivariate regression analyses showed that age, HCY and TC levels, undergoing glycemic control and the presence of CKD were positively associated with serum BUN levels in males with hypertension. LVEF, the presence of stroke and alcohol consumption were not independent factors to predict serum BUN levels in males. Age and the presence of CKD were positively associated and LDL-C levels and LVEF were negatively associated with serum BUN levels in females with hypertension. HCY levels, DBP, undergoing glycemic control, and the presence of CHD and stroke were not independent risk factors associated with serum BUN levels in females with hypertension.

**Table 4:** Factors associated with BUN levels in the multivariate regression analysis.

Variable	Male		Female	
	(N = 323)	*P*	(N = 177)	*P*
Age, years	0.05 (0.02 to 0.07)	< 0.001	0.04 (0.01 to 0.07)	0.025
HCY, μmol/L	0.07 (0.02 to 0.11)	0.006	–	–
TC, mmol/L	0.61 (0.30 to 0.93)	< 0.001	–	–
LDL-C, mmol/L	–	–	− 0.39 (− 0.70 to − 0.08)	0.016
LVEF, %	–	–	− 0.10 (− 0.16 to − 0.03)	0.004
Diabetes treatment, cases (%)	1.46 (0.81 to 2.11)	< 0.001	–	–
Presence of CKD, cases (%)	3.89 (2.94 to 4.83)	< 0.001	6.15 (4.71 to 7.60)	< 0.001

For the abbreviations, please see Table 1. Associations are expressed as coefficients and 95% CIs. "–" indicates that a variable is not an independent one in a given multivariate analysis model (p > 0.05).

### Factors independently associated with serum uric acid levels

As shown in Table 5, when age, BMI, HCY, HbA1c, LDL-C, and HDL-C levels and the presence of diabetes, CKD and stroke were inputted into the multivariable regression model, only BMI and presence of CKD remained as independent risk factors positively associated and HbA1c and HDL-C levels and the presence of stroke remained as independent risk factors negatively associated with serum uric acid levels in males with hypertension. Of females with hypertension, among the potential risk factors, age, HCY and HDL-C levels, LVEF, the presence of CKD and stroke, only LVEF and presence of CKD remained as independent predictors that were negatively and positively associated with serum uric acid levels.

**Table 5:** Factors associated with serum uric acid levels in the multivariate regression analysis.

Variable	Male		Female	
	(N = 323)	*P*	(N = 177)	*P*
BMI	5.59 (1.82 to 9.36)	0.004	–	–
HbA1c, %	− 0.13 (− 0.20 to − 0.06)	< 0.001	–	–
HDL-C, mmol/L	− 0.50 (− 0.88 to − 0.13)	0.008	–	–
LVEF, %	/	–	− 4.03 (− 7.02 to − 1.05)	0.008
Presence of CKD, cases (%)	80.20 (48.13 to 112.27)	< 0.001	108.85 (50.25 to 167.46)	< 0.001
Presence of stroke, case (%)	− 25.50 (− 47.24 to − 3.76)	0.022	–	–

For the abbreviations, please see Table 1. Associations are expressed as coefficients and 95% CIs. "–" indicates that a variable is not an independent one in a given multivariate analysis model (p > 0.05).

## Discussion

Our data showed that HCY levels were independent predictors positively associated with increased serum creatinine and BUN levels only in male patients. Since the radioenzymic determination showed that only tiny amounts of homocysteine are excreted in the urine^3,25^ and serum creatinine and BUN levels are the two main parameters that reflect renal capacity to eliminate metabolic byproducts, the higher serum homocysteine, the higher creatinine and BUN levels in male patients with hypertension should be caused by distinct mechanisms. Most likely, the former is caused by impaired nonrenal disposal, and the latter is a consequence of reduced renal elimination. Together with the biotoxicity of homocysteine described in the “Introduction”^1^, disorders in homocysteine metabolism might cause direct damage to the kidneys. Studies have showed that imbalance in homocysteine metabolism lead to molecular and cellular damage on many organs via homocysteinylation, oxidative stress induction, and excitotoxicity^1–3,7,19^. High homocysteine level in old male patients with hypertension may also be harmful to renal corpuscles, renal tubules and renal artery through the above mechanism. On the other hand, high circulating homocysteine levels might also lead to secondary renal injury via other complications such as hypertension^2^, etc.

In terms of why the above results were not observed in females with hypertension, numerous reports are consistent with our results and provide possible mechanism^26,27^. Firstly, different sex hormone levels between men and women may affect the metabolism of homocysteine^26,27^; the Third National Health and Nutrition Examination Survey stated that “…higher estrogen status is associated with a decreased mean serum total homocysteine concentration…” ^28,29^. Secondly, the serum homocysteine concentration in smokers is higher than that in non-smokers^30^; in our subjects, 34.5% males smoke, in contrast, only 1.7% females smoke. Finally, the level of the homocysteine in the serum is influenced by the presence of folic acid, vitamins B6 and B12^31^; the folic acid level of our female subjects is 9.2 ± 4.7 ng/mL, which is higher than that of males (7.6 ± 4.6 ng/mL).

Different from serum creatinine and BUN levels, serum uric acid levels are determined by the balance between dietary intake, endogenous metabolism of purines and the urinary excretion rate^32^; higher serum uric acid levels are always caused by overeating. Homocysteine is neither an independent risk factor for uric acid in males nor in females, which suggests that homocysteine is associated with renal damage through a pathway different from that involved in metabolic disorders.

The serum lipid representative LDL-C was an independent risk factor that was positively associated with increased serum creatinine levels only in male patients and negatively associated with increased serum BUN levels only in female patients. Although this content was not the focus of this study, the research available for reference is also very limited^33^. LDL-C levels were reported to be lower or higher in CKD patients^34,35^; since high LDL-C levels are a cause of atherosclerosis, it is understandable that LDL-C is positively associated with serum creatinine levels. What is puzzling is that LDL-C was negatively associated with BUN levels in female patients. A possible explanation for these results is sex differences and a discrepancy in LDL-C and BUN in their respective metabolic pathways. Reduced protein intake in our female subjects might be a reason why the higher the LDL-C levels were, the lower the BUN levels. HDL-C levels were independent risk factors that were negatively associated with increased serum uric acid levels only in male patients. Studies have shown that lower plasma apoA-I and plasma lecithin-cholesterol acyltransferase activity are present in subjects with chronic renal failure and lead to impaired HDL-mediated reverse cholesterol transport^33,36,37^. Although only 13.4% of male patients and 7.3% of female patients had CKD, the above mechanism could partially explain the associations of HDL-C in our report. In addition, negative associations between HDL-C and uric acid levels were also observed in asymptomatic dyslipidemic subjects^38^, which is also partially consistent with our results.

Age was a common independent risk factor that was positively associated with serum BUN levels in both sexes and was independently associated with serum creatinine levels in female patients. Age and sex are known to be two critical factors to estimate the glomerular filtration rate^39^; thus, the fact that age is associated with renal function is consistent with existing scientific knowledge. In terms of why age is not an independent factor associated with serum creatinine levels in males, this might be due to the particularity of age composition in our subjects between sexes.

LVEF was independently and negatively associated with serum BUN and uric acid levels in female patients and serum creatinine levels in male patients. Renal function disorders share many risk factors for CHD and hypertension^40^; thus, although the associations differed by sex and renal function indicators, our data suggest that the higher the serum creatinine, BUN and uric acid levels are, the lower the LVEF, which is in line with biomedical principles.

Regarding medical history, glycemic control was positively associated and the presence of stroke was negatively associated with serum BUN and uric acid levels in males with hypertension. The lower uric acid levels in male patients with hypertension and stroke might be due to diet control among these subjects. Other variables, such as smoking, alcohol consumption and taking medications for hypertension and/or CHD were not associated with renal function in males even in the preliminary univariate regression analyses; this might be due to the relatively low role of these factors in renal damage in older patients with hypertension.

In addition to acting as a renal function indicator, serum uric acid levels are also a main parameter for metabolic disorders^41^. BMI and HbA1c emerged as risk factor that were independently associated with serum uric acid levels in males with hypertension, which is in line with the above principles and the metabolic characteristics of our subjects that were summarized in the Table 1.

It is worth emphasizing that our study subjects were older Chinese adults with hypertension; our analytic strategy treated renal function indicators as continuous variables; only 13.4% of males and 7.3% of females were diagnosed with CKD, and our study quantified the relationship between biochemical indexes, including homocysteine levels and renal function but not the presence of CKD in adults with hypertension. Since hypertension, CKD, stroke and CHD are highly prevalent in older Chinese individuals, it is impossible to assemble enough age-matched and complication-free controls to rule out the association between homocysteine levels and renal function or hypertension in clinical practice.

In conclusion, our data indicate that in older Chinese males with hypertension, homocysteine levels are independent risk factors that are positively associated with increased serum creatinine and urea nitrogen levels. Our data imply that clinicians need to be aware of any possible clinical and subclinical renal injury caused by higher homocysteine level in old males with hypertension. Longitudinal cohort study is needed to evaluate a causal relationship between higher homocysteine level and renal damage in this population. Also, study to evaluate whether lowering homocysteine intervention (e.g. using folic acid and B vitamins) could improve renal function in older males with hypertension has clinical significance.
